# KISS1R and ANKRD31 Cooperate to Enhance Leydig Cell Gene Expression *via* the Cytoskeletal-Nucleoskeletal Pathway

**DOI:** 10.3389/fcell.2022.877270

**Published:** 2022-06-23

**Authors:** Giulia Ricci, Florian Guillou, Angela Catizone, Vincenza Grazia Mele, Martina Moggio, Teresa Chioccarelli, Nadia Diano, Rosaria Meccariello, Riccardo Pierantoni, Silvia Fasano, Gilda Cobellis, Rosanna Chianese, Francesco Manfrevola

**Affiliations:** ^1^ Dipartimento di Medicina Sperimentale, Università degli Studi della Campania L. Vanvitelli, Naples, Italy; ^2^ CNRS, IFCE, INRAE, Université de Tours, PRC, Nouzilly, France; ^3^ Dipartimento di Scienze Anatomiche, Istologiche, Medico Legali e dell’Apparato Locomotore, “Sapienza” Università di Roma, Roma, Italy; ^4^ Dipartimento di Scienze Motorie e del Benessere, Università di Napoli Parthenope, Napoli, Italy

**Keywords:** kisspeptin, KISS1R, ankyrins, male fertility, Leydig cells, cytoskeletal–nucleoskeletal pathway, actin

## Abstract

Kisspeptins are involved in the regulation of hypothalamic-pituitary-gonadal axis, Leydig cell functions, and testosterone secretion, acting as endogenous ligands of the KISS1 receptor. ANKRD31 protein participates in male fertility, regulating meiotic progression, and epididymal sperm maturation. Here, we show that in Leydig cells, KISS1 receptor and ANKRD31 proteins physically interact; the formation of this protein complex is enhanced by Kisspeptin-10 that also modulates F-actin synthesis, favoring histone acetylation in chromatin and gene expression *via* the cytoskeletal–nucleoskeletal pathway. Kp/KISS1R system deregulation, expression impairment of cytoskeletal–nucleoskeletal mediators, Leydig gene targets, and the decreased testosterone secretion in *Ankrd31* ^*−/−*^ testis strongly supported our hypothesis. Furthermore, cytochalasin D treatment subverted the gene expression induction dependent on Kisspeptin-10 action. In conclusion, the current work highlights a novel role for the Kisspeptin-10 in the induction of the cytoskeletal–nucleoskeletal route, downstream a physical interaction between KISS1 receptor and ANKRD31, with gene expression activation as final effect, in Leydig cells.

## Introduction

Kisspeptins (Kps), encoded by *Kiss-1* gene, are a group of neuropeptides involved in the neuroendocrine control of reproduction because they are able to stimulate the secretion of gonadotrophin-releasing hormone (GnRH), luteinizing hormone (LH), and follicle-stimulating hormone (FSH), through the activation of a G protein-coupled receptor, KISS1R (Oakley et al., 2009; Pinilla et al., 2012).

The action of Kps on GnRH neurons is tightly linked to the expression of KISS1R receptor on themselves (Colledge, 2009). KISS1R activation, dependent on Kisspeptin binding, is able to induce a potent GnRH neuron depolarization, associated with an intrinsic Kp-dependent gonadotrophin-releasing regulation, electing so, the Kp/KISS1R system act as a key mediator in the regulation of hypothalamic-pituitary-gonadal (HPG) axis at puberty and during adulthood (Thompson et al., 2004; Han et al., 2005; Navarro and Tena-Sempere, 2011). Indeed, Kps stimulate GnRH secretion, associated with a prominent LH-releasing downstream effect, in several species, including rodents and primates (Shahab et al., 2005; Chianese et al., 2016). Accordingly, the lack of Kp signaling results in hypogonadotropic hypogonadism and delayed sexual maturation (de Roux et al., 2003; Seminara et al., 2003; León et al., 2016). Despite the primary effect of Kps in the regulation of the HPG axis at the central level, a peripheral expression of the Kp/KISS1R system in mammalian and non-mammalian gonads has been reported (Gaytan et al., 2007; Chianese et al., 2013, 2015, 2017; Calder et al., 2014; Dorfman et al., 2014; Salehi et al., 2015; Meccariello et al., 2020; Gloria et al., 2021), although its testicular biological role needs to be further explored (Sharma et al., 2020). In this regard, recent findings support the involvement of Kps in Leydig cell functions, including testosterone secretion (Salehi et al., 2015). In particular, progressive Kp expression during mouse pubertal development, especially in Leydig cells, confirms a testicular local effect of Kps. In addition, isolated Leydig cells, *in vitro* treated with Kps, show enhanced KISS1/KISS1R gene expression and testosterone secretion, suggesting an autocrine role of the Kp/KISS1R system in these cells (Han et al., 2020).

Ankyrin repeat domain-containing proteins (ANKRDs) are sub-membranous proteins that favor the direct interaction between membrane- and cytoskeletal proteins, acting as a link-protein scaffold (Batrukova et al., 2000; Hryniewicz-Jankowska et al., 2002; Manfrevola et al., 2021a). This intricate physical protein interaction regulates several biological functions, such as cellular adhesion and cytoarchitecture (Cunhaa and Mohler, 2009). The favorite ANKRD cytoskeletal protein interactors are the SPECTRINS and, in turn, the actins, through which mechanobiological pathways are drawn (Xu et al., 2013; Saito et al., 2015; Dou et al., 2018).

In particular, mechanotransduction pathways, through the formation of cytoskeletal–nucleoskeletal connections, transduce extracellular mechanical forces into biochemical signals propagating them along the cytoskeleton to the nuclear envelope, in order to regulate chromatin organization and gene expression, as the final biological effect (Uhler and Shivashankar, 2017; Manfrevola et al., 2021a). New insights regarding the involvement of ANKRDs in mechanotransduction have been reported as ANKRDs, interacting with mechanosensitive channels, are required for the cytoplasmic transmission of mechanosensory activity in neurons (Argudo et al., 2019; Tang et al., 2020). In addition, ANKRD responsiveness to Kp/KISS1R signaling has been unveiled in GnRH neurons (Soga et al., 2016).

Interestingly, an emerging role of ANKRDs in male reproduction has also been reported. Recent studies have highlighted the involvement of ANKRD31 in male sterility, focusing the attention on the main role of ANKRD31 in homologous recombination, meiosis progression, blood–epididymal barrier integrity, and epididymal sperm maturation (Boekhout et al., 2019; Acquaviva et al., 2020; Manfrevola et al., 2021b).

A putative involvement of ANKRD31, in mediating Kp signaling in the regulation of Leydig cell functions and gene expression, through the cytoskeletal–nucleoskeletal pathway, is a novel aspect investigated here.

In the current work, we report the testicular responsiveness to Kp/KISS1R system stimulation, through the analysis of cytoskeletal–nucleoskeletal modulators and Leydig gene targets. In detail, we confirm the expression of the Kp/KISS1R system in murine Leydig cells, and we show that Kisspeptin-10 (Kp-10), *via* KISS1R activation, induces the expression of KISS1R and ANKRD31, with an upregulation of cytoskeletal–nucleoskeletal actors. Testicular KISS1R activation promotes the expression of Leydig cell genes, a biological effect that we found correlated with an increasing histone acetylation.

Based on these observations, we hypothesized that the Kp/KISS1R system could regulate Leydig cell functions and gene expression *via* ANKRD31 and the cytoskeletal–nucleoskeletal pathway. In support to this hypothesis, we demonstrate a complete Kp/KISS1R system deregulation in *Ankrd31* ^*−/−*^ testis associated with the expression impairment of cytoskeletal–nucleoskeletal mediators, as well as of Leydig gene targets and testosterone secretion. In addition, we show, for the first time, the interaction among KISS1R, ANKRD31, and F-actin proteins in murine primary Leydig cell cultures. Furthermore, the stimulation of Leydig cells with Kp-10 induces F-actin synthesis and, sequentially, enhances nuclear histone acetylation with a subsequent increase in gene expression, leading us to suppose a direct involvement of F-actin and the cytoskeletal–nucleoskeletal pathway in this biological effect. Cytochalasin D treatment carried out in primary Leydig cell cultures subverted the Kp-dependent induction of both histone acetylation and Leydig gene expression, confirming that Kp-10 enhances KISS1R/ANKRD31 interaction and, in turn, cytoskeletal–nucleoskeletal actors, in order to remodel chromatin and regulate Leydig gene expression.

## Materials and Methods

### Experimental Animals

C57BL/6 male mice (Charles River Laboratory, Lecco, Italy) were used in this study. All animals were housed as three per cage under controlled illumination (12 h light/dark cycle; light on 6:00 a.m.) and standard environmental conditions (ambient temperature 20–22°C, humidity 55%–60%) and were maintained on a standard pellet diet with free access to water, before the beginning of experimental procedures. The number of the enrolled animals was determined by the parameters adopted for the G*Power analysis required to get the permission for *in vivo* experiments, which is suggested by the legal entity giving the permission. For experimental procedures, adult males (3–5 months) were sacrificed under anesthesia, by cardiac perfusion with PBS (pH 7.6), to clean peripheral tissues from blood contaminants, or by cervical dislocation, depending on the experimental designs. Animals were placed in a plexiglas chamber with 4% isoflurane (Iso-Vet, Piramal Healthcare, United Kingdom) for 5 min and were sacrificed when fully sedated, as measured by a lack of heartbeat and active paw reflex. Testes were rapidly removed and collected depending on the experimental procedure, as described later.

In addition, wild-type (WT) male mice and males carrying *Ankrd31* null mutation (*Ankrd31* ^−/−^) (Manfrevola et al., 2021b) were used in this study. Heterozygous mice were bred on a C57BL/6 background before generating WT and *Ankrd31* ^−/−^ male mice. For experimental procedures, WT and *Ankrd31* ^−/−^ adult males (3–5 months) were sacrificed, under anesthesia, by cervical dislocation. Testes and blood samples were rapidly removed and collected depending on the experimental procedures.

### Chemicals

Kp-10 (Metastin 45–54, H-YNWNSFGLRF-NH2) of human origin and Kp-234, a specific KISS1R antagonist (Roseweir et al., 2009), were purchased from DBA Italia (Milan, Italy). Kp-10 of human origin was chosen on the basis of its high homology with the murine peptide sequence. Cytochalasin D (C8273) was obtained from Sigma-Aldrich (Milan, Italy). The drugs were dissolved in dimethylsulfoxide (DMSO), according to the manufacturer’s instructions. NB4 collagenase was obtained from Serva (Heidbergh, Germany).

### Histology and Immunocytochemistry Analysis

Testes collected from WT and *Ankrd31* ^*−/−*^ mice (n = 5 for each group) were fixed overnight in Bouin’s solution, dehydrated in ethanol, cleared in xylene, and embedded in paraffin using standard procedures. Microtome serial sections (7 μm thick) were cut and processed for hematoxylin/eosin (H&E) staining and immunocytochemistry staining analyses. For H&E staining, the sections were deparaffinized and processed using standard procedures. For immunocytochemistry staining, testes sections were deparaffinized, rehydrated, and permeabilized with PBS pH 7.4 containing 0.1% Triton X-100. A citrate buffer of 0.01 M (pH 6.0) was used for antigen retrieval. After blocking with PBS containing 5% BSA and normal goat serum (diluted 1:5), sections were incubated with anti-KISS1 antibody (PA5-50513, Invitrogen; diluted 1:100), anti-KISS1R antibody (PA5-96221, Invitrogen; diluted 1:100), anti-F-actin antibody (MA1-80729, Invitrogen; diluted 1:100), anti-H3K14ac antibody (703894, Invitrogen; diluted 1:100), and anti-StAR antibody (sc-166821, Santa Cruz, diluted 1:100) overnight at 4°C. Immunoreactivity was revealed using the avidin/biotin complex system and H_2_O_2_/DAB as the substrate/chromogen. The specificity of immunoreactions is routinely checked by omitting primary antibodies. The histological observations and analysis were carried out under a light microscope (Leica CTR500, Leica Microsystems Inc., Milan, Italy), and images were captured using a high-resolution digital camera (Leica DC300F).

### Human Chorionic Gonadotropin (hCG) Administration *in vivo*

WT (n = 5) and *Ankrd31* ^*−/−*^ (n = 5) male mice (3 months old) were injected with hCG (15 IU/Kg; Chorulon Grovet) intraperitoneally. After 2 h from hCG injection, blood samples were collected and plasma was obtained by centrifugation at 1,000× g for 10 min. Plasma samples were stored at −20°C for further testosterone dosage.

### Testosterone EIA Assay

Plasma TT levels of WT (n = 5) and *Ankrd31* ^*−/−*^ (n = 5) male mice, at basal conditions and following hCG injection, were measured by a competitive enzyme immunoassay (EIA) designed by the Phenotyping Endocrinology Laboratory (Research unit: Physiology of Reproduction and Behavours, Nouzilly, France). The sample size used for the assay was 8 µL of undiluted sample. The minimal detectable TT concentration was 0.15 ng/ml, and all samples were analyzed in the same assay. The intra- and inter-assay coefficients of variation were 4.7% and 7.1%, respectively. All determinations were made in triplicate for each plasma sample analyzed.

### Kp-10 *In Vitro* Treatment of Mouse Testis

C57BL/6 testes (n = 5 for each experimental group) were incubated in PBS for 90 min at room temperature (RT), with vehicle (0.005% DMSO; control group, CTRL) or with Kp-10 at 0.01 μM, 0.1 μM, and 1 μM. After treatment, the testes were placed at −80°C for following molecular investigations.

### Isolation and Culture of Mouse Leydig Cells

Leydig cells were isolated as previously described by Sun et al. (2011). In brief, testes from adult mice (n = 5) were harvested, and tunica albuginea was removed. Then, testes were digested at 37°C for 15 min in 0.03% collagenase NB4 in a thermomixer imposing 150 rpm vibration. After this first digestion, the supernatant was discarded. Then, testes were incubated again in 0.03% collagenase NB4 in the thermomixer for 15 min, lowering the vibration speed to 130 rpm. Isolated cells were counted and approximately 1 × 10^5^ cells per testis were obtained. Isolated cells were cultured in low glucose DMEM with 10% of fetal bovine serum (FBS) plating them in culture plates (60 mm or 35 mm culture dish Falcon) for molecular analysis and in eight well chamber slides (Ibidi) for immunofluorescence experiments. STAR staining was carried out to identify the cells and to assess their purity (data not shown). When the Leydig cells were grown to 90% confluence were treated, when indicated, with Kp-10 (0.1 µM), Kp-234 (1 µM), and cytochalasin D (10 µM) alone or in combination with Kp-10. Cytochalasin D and Kp-234 were added 30 min before Kp-10 treatment.

### Immunofluorescence and Confocal Microscopy Analysis

For *in situ* analyses, Leydig cells, cultured in eight well chamber slides, were fixed in 4% paraformaldehyde in PBS (pH 7.4) at 4°C overnight. To detect F-actin and H3K14ac, immunofluorescence experiments were performed. Fixed cells were permeabilized in PBS supplemented with 1% BSA and 0.1% Triton for 2 h and then quenched with 5% donkey serum in PBS/BSA/Triton. Samples were then incubated overnight with anti-H3K14ac antibody diluted 1:100 (703894, Invitrogen Life Technologies, Paisley, United Kingdom). Then, samples were washed three times in PBS/BSA/Triton for 30 min and incubated with the appropriate secondary antibody: Cy5 conjugated anti-rabbit IgG was used to detect H3K14ac immunocomplexes (Jackson Immuno Research, Cambridge, United Kingdom). TO-PRO3 iodide fluorescent dye 642/661 (1:5000 in PBS, Invitrogen Life Technologies, Paisley, United Kingdom) was used for nuclei staining. As a negative control, the primary antibody was omitted. For the detection of F-actin, a FITC-conjugated phalloidin (Invitrogen, Life Technologies, Paisley, United Kingdom) was used.

Immunofluorescence experiments were analyzed using a Leica confocal microscope (Laser Scanning TCS SP2 equipped with Kr/Ar and He/Ne lasers, Mannheim, Germany). Laser lines were 488, 543, and 633 nm for FITC, Cy5, and TO-PRO3 excitation, respectively. The images were scanned under a ×20 or ×40 oil immersion objectives. To perform quantitative analysis of fluorescence, optical spatial series with a step size of 1 µm were recovered with fixed laser intensities, and the sum of fluorescence intensity (SUM(I)) was determined in maximum projection image of each series using Leica confocal software. Three independent experiments in triplicate were analyzed.

### Determination of TT Levels in Leydig Cell Culture Media

Culture media of Leydig cells *in vitro* treated with Kp-10 (0.1 µM) alone or in combination with Kp-234 (1 µM) were collected to perform TT determination. TT was extracted from cell culture media using methanol as an organic solvent (liquid/liquid extraction) and purified by solid phase extraction (SPE) using specific cartridges for steroids (AFFINIMIP, Affinisep, France). The procedure described by Errico et al. (2019) was modified specifically for cell culture media including the quality control system used to monitor method performance and to prevent assay contamination. The LC-MS/MS analysis was performed using a Dionex UltiMate3000 HPLC system (Thermo Fisher Scientific Inc, Monza, Italy), coupled to an ESI-triple quadrupole mass spectrometer (API 2000, Sciex, Germany). A Kinetex F5 (×100 4.6 mm, 2.6 µm) column (Phenomenex, Italy) was used for reversed-phase separations. Chromatographic separation and instrumental parameters were reported in Errico et al., 2019. The analyte identification was based on multiple reactions monitoring in a positive mode. The TT was specifically identified not only by the retention time but also by monitoring the following ion transitions: m/z 291.3 → m/z 97.2 (quantifier) and m/z 291.3 → m/z 108.9 (qualifier). The linearity of the detector response was verified over the concentration range 0.100–50 ng/ml. All samples were analyzed in triplicate, for each experimental group, with relative standard deviations (RSDs) less than 13%. HPLC grade reagents, including ultrapure water, acetonitrile (ACN), and methanol (MeOH), were purchased from Romil (ROMIL Ltd., United Kingdom). TT was purchased from Merch (Germany). AFFINIMIP cartridges were purchased from Affinisep (France).

### Total RNA Preparation

The TRIzol^®^ reagent (Invitrogen Life Technologies, Paisley, United Kingdom) was used to extract total RNA from C57BL/6 testes *in vitro* treated with Kp-10 (0.01 μM, 0.1 μM, and 10 μM) and from primary murine Leydig cells *in vitro* treated with Kp-10 (0.1 μM) and cytochalasin D (10 μM) alone or in combination with Kp-10 (0.1 μM). In brief, the testes were homogenized in the TRIzol reagent and incubated for 5 min at 20°C. Then, 0.2 ml chloroform/ml Trizol reagent were added, and the samples were centrifuged at 12,000× g for 15 min at 4°C. The aqueous phase was transferred to a fresh tube and total RNA was precipitated by mixing with isopropyl alcohol (0.5 ml/ml Trizol reagent) and 1 μl of glycogen (20 mg/ml). After centrifugation at 12,000× g for 10 min at 4°C, the RNA pellet was washed with 75% ethanol, centrifuged at 7,500× g for 10 min at 4°C, and dissolved in DEPC-H_2_O. Total RNAs were assessed with a NanoDrop 2000 spectrophotometer (Thermo Fisher Scientific, Waltham, MA, United States) to quantify concentration (ng/μl) and purity (260/280 and 260/230 ratios). Then, RNA aliquots (10 μg) were treated with 2U DNase I (RNase-free DNase I, Ambion, Thermo Fisher Scientific, Massachusetts, United States) to remove potential contamination of genomic DNA and finally preserved at −80°C until the next step.

### RNA Expression Analysis by One-Step Evagreen qRT-PCR

According to the manufacturer’s instructions, a kit containing quantitative real-time polymerase chain reaction (qRT-PCR) enzyme mix and an Evagreen qPCR Mastermix (Applied Biological Materials Inc., Richmond, Canada) was used for gene expression analysis in i) testes *in vitro* treated with Kp-10 (0.01 μM, 0.1 μM, and 10 μM) (n = 5 animals for each experimental group) and in ii) primary murine Leydig cells *in vitro* treated with Kp-10 (0.1 μM) and cytochalasin D (10 μM) alone or in combination with Kp-10 (0.1 μM) (n = 5 samples for each experimental group). A concentration of 50 ng of total RNA was used for all reactions on a CFX-96 Real-Time Polymerase Chain Reaction (PCR) System (Biorad, Milan, Italy). A negative control, without RNA, was included. The qRT-PCR in triplicate from each experimental group was analyzed. A gene expression analysis, corrected for PCR efficiency, and normalized toward the reference gene (*Rp18S*), was performed by CFX Manager software (Biorad, Milan, Italy). Normalized fold expression (n.f.e) of mRNAs was calculated by applying the 2^–ΔΔCT^ method.

### PCR Primer Design

Primers to amplify selected RNAs were designed through the online tool Primer-BLAST1. Primers for mouse genes are shown in Table 1.

**TABLE 1:** Primer sequences and annealing temperatures.

Gene primers	Sequences 5′–3′	Tm (°C)
*Ankrd31*S	CAT​ATA​TGC​TAA​TGG​TAC​CCT​ACC​A	53
*Ankrd31* AS	CCT​TGT​AAT​TAG​TAA​TTT​GCC​ACA​G	
*KISS1R* S	GCC​ACA​GAC​GTC​ACT​TTC​CTA​C	55
*KISS1R* AS	CGG​GAA​CAC​AGT​CAC​ATA​CCA	
*Spectrin* S	ACT​TGG​AGC​AGG​TTG​AGG​TG	57
*Spectrin* AS	TGC​ACT​TCC​TCT​GCC​ATC​AG	
*Nesprin2* S	CGA​GCT​GGA​AGC​TCT​GAA​GT	58
*Nesprin2* AS	ATG​GAG​TCT​ATT​TTG​GAG​TTC​TGT​G	
*Sun2* S	CAC​TCG​CTA​CTC​TCA​GGA​TGA​TAA	52
*Sun2* AS	TAG​GAC​TCT​CGA​ACC​ACA​GAC​TC	
*Star* S	GGC​CAC​ACA​TTT​TGG​GGA​GA	56
*Star* AS	GGC​GAA​CTC​TAT​CTG​GGT​CTG	
*Lhr* S	GGG​CTG​GAG​TCC​ATT​CAG​AC	58
*Lhr* AS	CAC​AGC​AGT​GGC​TAG​GGT​AG	
*Sf1* S	GTG​TAC​CAA​GTG​TGG​AGG​GG	55
*Sf1* AS	CAC​AGA​TGC​AGG​GAC​AGG​AG	
*Hsd3b* S	TGT​GCA​TTA​AGG​CCC​ATG​TTT	52
*Hsd3b* AS	TTG​AGG​GCC​GTA​ATT​ATT​GTG​TT	
*Actb* S	GGC​TGT​ATT​CCC​CTC​CAT​CG	55
*Actb* AS	CCA​GTT​GGT​AAC​AAT​GCC​ATG​T	
*Rp18 S* S	GAG​ACT​CTG​GAT​GCT​AAC​TAG	56
*Rp18S* AS	GGA​CAT​CTA​AGG​GCA​TCA​CAG	

### Protein Extraction and Western Blot Analysis

Total protein extraction was performed for i) testes *in vitro* treated with Kp-10 (0.01 μM, 0.1 μM, and 10 μM) (n = 5 animals for each experimental group), ii) primary murine Leydig cells *in vitro* treated with Kp-10 (0.1 μM) alone or in combination with the specific antagonist Kp-234 (1 μM) (n = 5 samples for each experimental group), and iii) primary murine Leydig cells *in vitro* treated with Kp-10 (0.1 μM) and cytochalasin D (10 μM) alone or in combination with Kp-10 (0.1 μM) (n = 5 samples for each experimental group). The samples were separately homogenized in RIPA buffer [PBS, pH 7.4, 10 mM of dithiothreitol, 0.02% sodium azide, 0.1% SDS, 1% NP-40, and 0.5% sodium deoxycholate, in the presence of protease inhibitors (10 μg/ml of leupeptin, aprotinin, pepstatin A, chymostatin, and 5 μg/ml of TPCK)] and sonicated three times for 30 s bursts, each at 60 mW. Proteins were separated by SDS-PAGE and transferred to a polyvinylidene difluoride membrane (GE Healthcare, Milan, Italy) at 280 mA for 2.5 h, at 4°C. The filters were treated for 2.5 h with blocking solution [5% non-fat milk, 0.25% Tween 20 in Tris-buffered saline (TBS, pH 7.6)] and incubated with different primary antibodies in TBS-milk buffer (TBS pH 7.6, 3% non-fat milk) overnight, at 4°C. The filters were washed in 0.25% Tween 20–TBS and incubated with secondary antibodies diluted 1:1,000 in TBS-milk buffer and then washed again. An enhanced chemiluminescence–Western blotting detection system (Amersham ECL Western Blotting Detection Reagent, cod: RPN2106, GE Healthcare, Milan, Italy) was used to detect the immune complexes.

Antibodies and relative dilutions are reported in Table 2. The specificity of the immunoreactions was routinely checked by omitting all primary antibodies used in this study (data not shown). Western blot experimental triplicates from each experimental group were quantified by densitometry analysis, adjusted relatively to Ponceau S staining, and reported as O.D. fold change (mean ± SEM).

**TABLE 2:** Primary antibodies, protein amounts, antibody dilution, and secondary antibodies used for Western blot analysis.

Primary antibody	µg of protein	Antibody dilution	Secondary antibody
KISS1R (Boster Bio, A01364-1)	50	1:500	HRP-conjugated rabbit IgG (Dako Corp., Milan, Italy)
ANKRD31 Manfrevola et al. (2021b)	60	1:500	HRP-conjugated rabbit IgG (Dako Corp., Milan, Italy)
NESPRIN2 (Invitrogen, PA5-78438)	50	1:500	HRP-conjugated rabbit IgG (Dako Corp., Milan, Italy)
SUN2 (Santa Cruz, sc-377459)	50	1:500	HRP-conjugated mouse IgG (Dako Corp., Milan, Italy)
SPECTRIN (Santa Cruz, sc-53444)	60	1:500	HRP-conjugated mouse IgG (Dako Corp., Milan, Italy)
HDAC1 (Santa Cruz, sc-8410)	60	1:500	HRP-conjugated mouse IgG (Dako Corp., Milan, Italy)
HDAC2 (Santa Cruz, sc-9959)	60	1:500	HRP-conjugated mouse IgG (Dako Corp., Milan, Italy)
HDAC4 (Santa Cruz, sc-46672)	60	1:500	HRP-conjugated mouse IgG (Dako Corp., Milan, Italy)
H3K14ac (Invitrogen, 703894)	50	1:500	HRP-conjugated rabbit IgG (Dako Corp., Milan, Italy)
β-actin (Invitrogen, PA1-183)	50	1:500	HRP-conjugated rabbit IgG (Dako Corp., Milan, Italy)
F-actin (Invitrogen, MA1-80729)	50	1:500	HRP-conjugated mouse IgG (Dako Corp., Milan, Italy)
LHR (Santa Cruz, sc-293165)	50	1:500	HRP-conjugated mouse IgG (Dako Corp., Milan, Italy)
HSD3β (Santa Cruz, sc-515120)	50	1:500	HRP-conjugated mouse IgG (Dako Corp., Milan, Italy)
SF1 (Santa Cruz, sc-393592)	50	1:500	HRP-conjugated mouse IgG (Dako Corp., Milan, Italy)
STAR (Santa Cruz, sc-166821)	50	1:500	HRP-conjugated mouse IgG (Dako Corp., Milan, Italy)
CYP19 [Invitrogen, PA1-21398)]	60	1:500	HRP-conjugated mouse IgG (Dako Corp., Milan, Italy)

### Protein Immunoprecipitation Assay

For IP, primary murine Leydig cells *in vitro* treated with Kp-10 (0.1 μM) alone or in combination with the specific antagonist Kp-234 (1 μM) were lyzed in RIPA buffer, in the presence of protease inhibitors (10 μg/ml of leupeptin, aprotinin, pepstatin A, chymostatin, and 5 μg/ml of TPCK), sonicated three times for 30 s bursts, each at 60 mW, and then incubated on ice for 30 min. After centrifugation at 20,000× g for 30 min at 4°C, the protein supernatant was collected. A concentration of 500 μg of supernatant proteins from each sample was incubated with 2 μg of KISS1R antibody (IP-KISS1R), ANKRD31 antibody (IP-ANKRD31), or IgG as negative control (12370, Sigma-Aldrich, Milan, Italy), under rotary agitation, overnight at 4°C. Afterward, Protein A/G PLUS Agarose Beads (sc-2003, Santa Cruz Biotechnology, Cambridge, United Kingdom) were added to each sample and incubated overnight under rotary agitation at 4°C. After bead incubation, samples were washed three times (3,000× g for 3 min at 4°C) in TBS pH 7.6 and boiled in Laemmli sample buffer for 10 min to be later analyzed by SDS-PAGE.

### Statistical Analysis

ANOVA followed by Student’s t-test (for two independent group comparisons) and Tukey test (for multi group comparison) was used to identify groups having different mean. Differences with *p* < 0.05 were considered statistically significant. Data were expressed as the mean ± SEM from at least five independent animals for each genotype or experimental group. For qRT-PCR, Western blot, LC-MS/MS, and EIA assay, triplicates from five animals/genotypes or experimental groups each were considered.

## Results

### Kp-10 Positively Regulates Testicular KISS1R Expression and the Cytoskeletal–Nucleoskeletal Pathway

**FIGURE 1:**
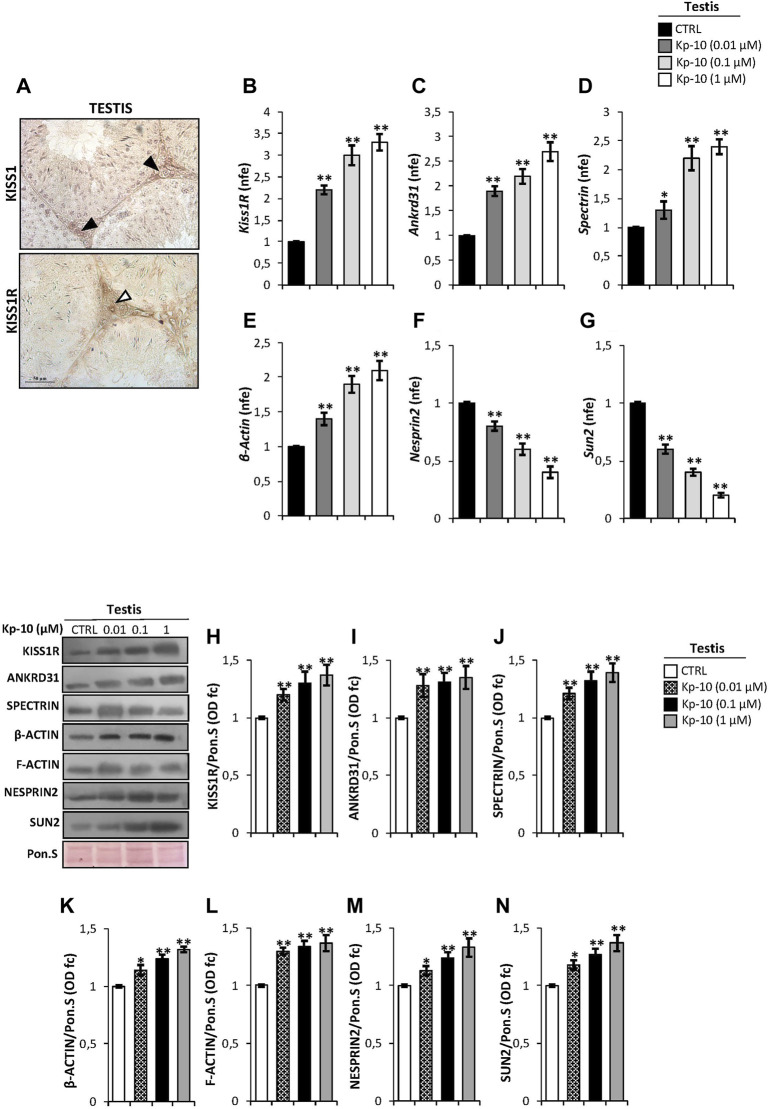
**(A)** Immunocytochemistry of KISS1 and KISS1R in Bouin’s fixed C57BL/6 testis sections (7 μm). The KISS1 and KISS1R protein localization in Leydig cells was indicated by black and white arrowheads, respectively. Scale bar: 50 μm. **(B–G)** Differential expression analysis of *KissR* and cytoskeletal–nucleoskeletal pathway mediator mRNAs in mice testes *in vitro* treated with different doses of Kp-10 (0.01 µM, 0.1 µM, and 1 µM), by qRT-PCR. **(B)** *Kiss1R*, **(C)** *Ankrd31*, **(D)** *Spectrin*, **(E)** *β-Actin*, **(F)** *Nesprin2*, and **(G)** *Sun2* expression levels were normalized using *Rp18S* as a housekeeping gene and expressed as normalized fold expression (n.f.e.), relatively to the CTRL group. All data are reported as mean value ± S.E.M; *p < 0.05; **p < 0.01. Western blot analysis of **(H)** KISS1R, **(I)** ANKRD31, **(J)** SPECTRIN-α-II, **(K)** β-actin, **(L)** F-actin, **(M)** NESPRIN2, and **(N)** SUN2 proteins levels in mice testes *in vitro* treated with different doses of Kp-10 (0.01 µM, 0.1 µM, and 1 µM). Signals were quantified by the densitometry analysis and normalized to Ponceau Red (Pon.S). Data are expressed in O.D. values as fold change (O.D. fc), relatively to the CTRL group, and reported as mean ± SEM; *p < 0.05; **p < 0.01.

In order to study the effects of Kps in the regulation of the Kp/KISS1R system and the cytoskeletal–nucleoskeletal pathway in Leydig cells, we evaluated i) the expression of the Kp/KISS1R system in WT mouse testis, by an immunocytochemistry analysis, and ii) the expression of several cytoskeletal–nucleoskeletal mediators, in testes *in vitro* treated with different doses of Kp-10 (0.01 µM, 0.1 µM, and 1 µM), by qRT-PCR. Results showed a striking KISS1 and KISS1R protein localization in Leydig cells (Figure 1A). Interestingly, higher expression levels of *Kiss1R*, at all the chosen doses of Kp-10, were observed in comparison to the CTRL group (Figure 1B) (*p* < 0.01). In addition, a significant increase in *Ankrd31*, *Spectrin*, and *β-Actin* expression levels was also observed at all the chosen doses of Kp-10, in comparison to the CTRL group (Figures 1C–E) (*p* < 0.05; *p* < 0.01), whereas lower expression levels of *Nesprin2* and *Sun2* were observed following Kp-10 *in vitro* treatment (Figures 1F,G) (*p* < 0.01).

Western blot analysis was carried out to investigate the testicular protein levels of KISS1R receptor and cytoskeletal–nucleoskeletal modulators, following Kp-10 *in vitro* treatment. As reported, data showed that all the analyzed proteins were significantly increased following Kp-10 treatment. In details, a significant increase of testicular KISS1R, ANKRD31, and SPECTRIN protein amount was observed at all the chosen doses of Kp-10, relatively to the CTRL group (Figures 1H–J) (*p* < 0.01). In agreement, the levels of cytoskeletal modulators, as β-actin and F-actin, were higher in testes *in vitro* treated with Kp-10 than the CTRL group (Figures 1K,L) (*p* < 0.05; *p* < 0.01) and, finally, a significant increase of NESPRIN2 and SUN2 protein levels was also observed at all the doses, relatively to the CTRL group (Figures 1M–N) (*p* < 0.05; *p* < 0.01), suggesting that Kp-10 *in vitro* treatment positively modulates the expression of both KISS1R receptor and cytoskeletal–nucleoskeletal pathway modulators, having Leydig cells as the main target.

### In Testis Kp-10 Enhances the Expression of Leydig Cell Genes and Epigenetic Markers

**FIGURE 2:**
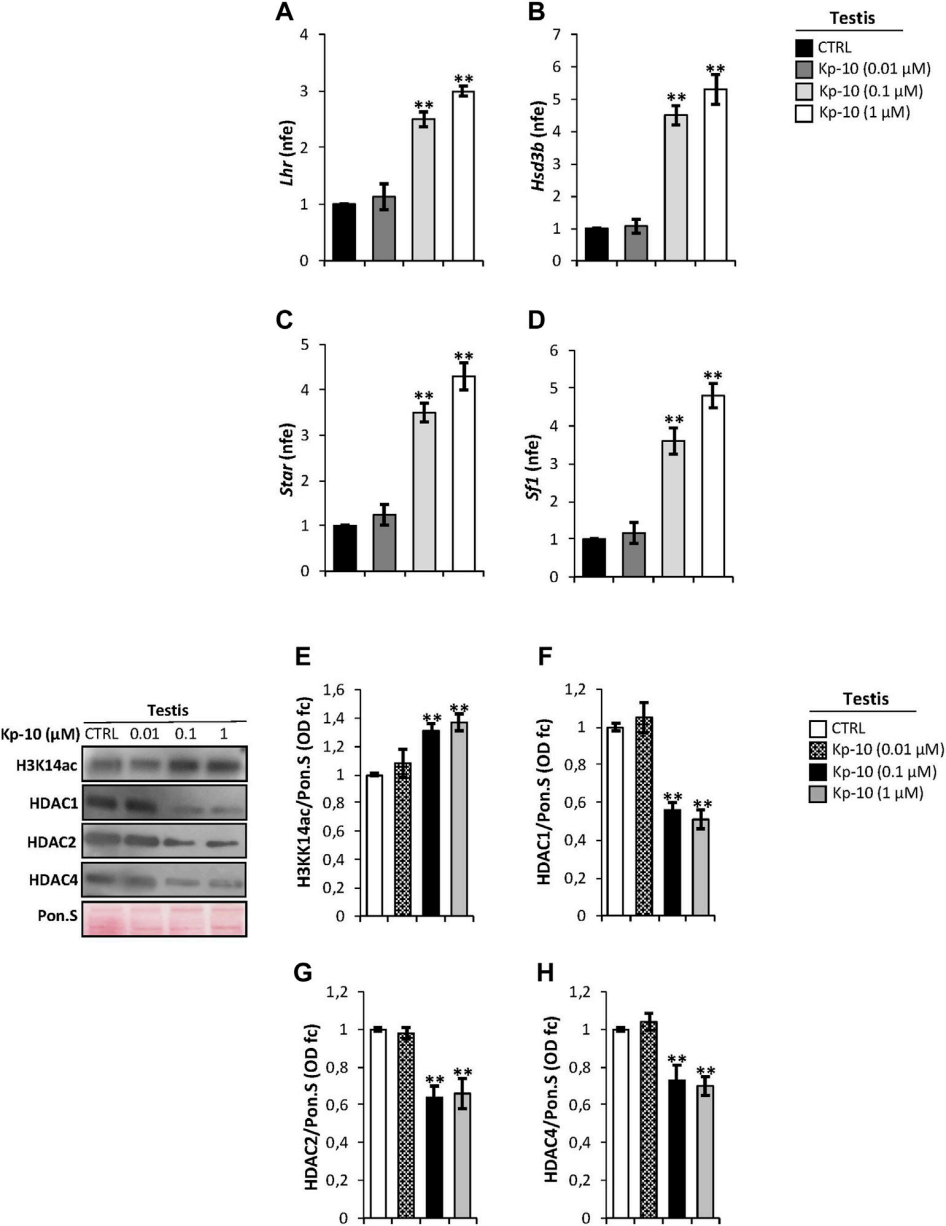
**(A–D)** Differential expression analysis of Leydig cell genes in mice testes *in vitro* treated with different doses of Kp-10 (0.01 µM, 0.1 µM, and 1 µM), by qRT-PCR. **(A)** *Lhr*, **(B)** *Hsd3b*, **(C)** *Star*, and **(D)** *Sf1* expression levels were normalized using *Rp18S* as a housekeeping gene and expressed as normalized fold expression (n.f.e.), relatively to the CTRL group. All data are reported as mean value ± S.E.M; **p < 0.01. Western blot analysis of **(E)** H3K14ac, **(F)** HDAC1, **(G)** HDAC2, and **(H)** HDAC4 protein levels in mice testes *in vitro* treated with different doses of Kp-10 (0.01 µM, 0.1 µM, and 1 µM). Signals were quantified by the densitometry analysis and normalized to Ponceau Red (Pon.S). Data are expressed in O.D. values as fold change (O.D. fc), relatively to the CTRL group, and reported as mean ± SEM; **p < 0.01.

Considering that in mouse testis KISS1R is expressed in Leydig cells (Anjum et al., 2012; Salehi et al., 2015), as confirmed by our immunocytochemistry analysis, we evaluated a possible effect of Kps on the expression of key modulators of steroidogenesis. Results showed a significant increase of *Lhr* and *Hsd3b* expression levels at the Kp-10 doses of 0.1 and 1 µM (*p* < 0.01), (Figures 2A,B), in comparison with the CTRL group, whereas no effect was observed at the Kp-10 dose of 0.01 µM (Figures 2A,B). Similar results were observed for *Star* and *Sf1* (Figures 2C,D).

Western blot analysis was carried out to investigate a possible responsiveness of epigenetic markers following Kp *in vitro* treatment. As showed, relatively to the CTRL group, a significant increase of H3K14ac protein levels, chosen as a marker of active gene transcription, was observed at Kp-10 doses of 0.1 µM and 1 µM (*p* < 0.01), whereas no effect was observed at the dose of 0.01 µM (Figure 2E). Analogously, testicular *in vitro* treatment with the doses 0.1 and 1 µM of Kp-10 significantly decreased protein levels of HDAC1, HADAC2, and HDAC4 (*p* < 0.01) than the CTRL group (Figures 2F,G), whereas no effect was observed at the dose of 0.01 µM for all HDAC types investigated, suggesting that Kps might positively regulate Leydig cell gene expression modulating epigenetic markers.

### *Ankrd31* Gene Deletion Negatively Affects Kp/KISS1R System and Leydig Functions

**FIGURE 3:**
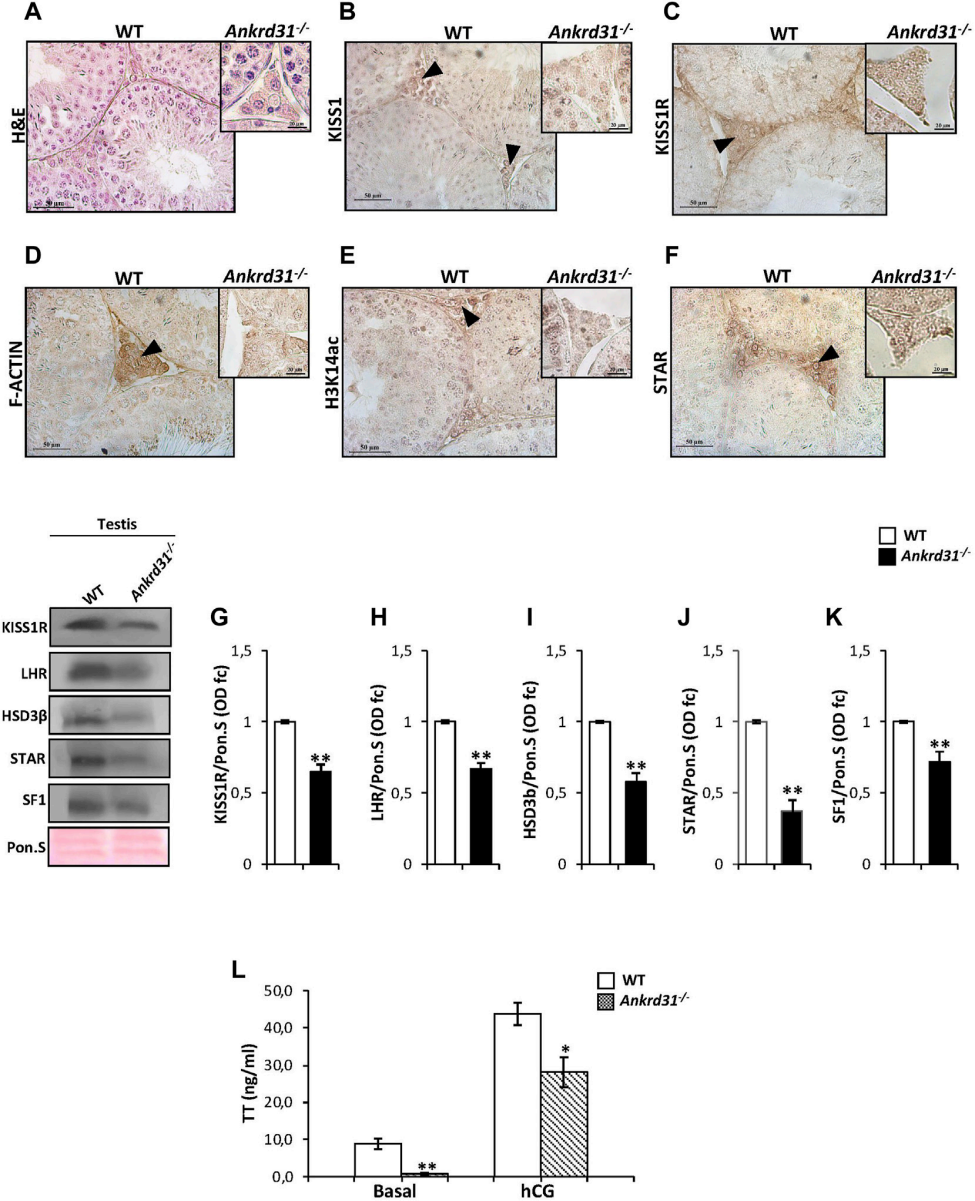
**(A)** H&E staining of Bouin’s fixed WT and *Ankrd31* ^*−/−*^ testes sections (7 μm). Leydig cells were indicated by black arrowheads. Scale bar: 50 μm. **(B–F)** Immunocytochemistry of **(B)** KISS1, **(C)** KISS1R, **(D)** F-actin, **(E)** H3K14ac, **(F)** STAR in Bouin’s fixed WT, and *Ankrd31* ^*−/−*^ testes sections (7 μm). The protein localization in Leydig cells was indicated by black arrowheads. Scale bar: 50 μm; scale bar inset: 50 μm. **(G–K)**. Western blot analysis of **(G)** KISS1R, **(H)** LHR, **(I)** HSD3β, **(J)** STAR, and **(K)** SF1 in WT and *Ankrd31* ^*−/−*^ testes. Signals were quantified by the densitometry analysis and normalized to Ponceau Red (Pon.S). Data were expressed in O.D. values as fold change and reported as mean ± SEM; **p < 0.01. **(L)** Plasma testosterone (TT) levels, at basal condition and following hCG stimulation, in WT and *Ankrd31* ^*−/−*^ mice by EIA assay; data were reported as mean ± SEM; **p < 0.01.

In order to demonstrate that the Kp/KISS1R system could regulate Leydig cell functions and gene expression *via* ANKRD31, and in turn by the cytoskeletal–nucleoskeletal pathway, we carried out immunocytochemistry and molecular analyses in *Ankrd31* ^*−/−*^ mouse testes, chosen as putative model of cytoskeletal disruption. A histological analysis performed by H&E staining on testicular sections of both WT and *Ankrd31* ^*−/−*^ showed an impairment of interstitial environment in *Ankrd31* ^*−/−*^ compared to WT (Figure 3A), suggesting that the loss of ANKRD31 protein could affect Leydig cell functions. Interestingly, the immunocytochemistry analysis showed a lower signal of both KISS1 and KISS1R in *Ankrd31* ^*−/−*^ Leydig cells than WT, confirming a deregulation of the Kp/KISS1R system (Figures 3B,C). In addition, the immunocytochemistry analysis of F-actin, H3K14ac as well as STAR evidenced a strong signal reduction of all markers analyzed in *Ankrd31* ^*−/−*^ compared to WT Leydig cells (Figures 3D–F). To confirm Kp/KISS1R system deregulation, the Western blot analysis of KISS1R was carried out in WT and *Ankrd31* ^*−/−*^ testis. As reported, a significant reduction of KISS1R protein content was observed in *Ankrd31* ^*−/−*^ in comparison to WT testis (*p* < 0.01) (Figure 3G). In order to assess if the deregulation of the Kp/KISS1R system observed in *Ankrd31* ^*−/−*^ Leydig cells occurred due to their compromised functions, we evaluated the expression of key modulators of steroidogenesis by the Western blot analysis. The results showed a significant decrease of LHR, HSD3β, STAR, and SF1 protein contents in *Ankrd31* ^*−/−*^ in comparison to WT (*p* < 0.01), (Figures 3H–K). In agreement, plasma TT levels were significantly lower in *Ankrd31* ^*−/−*^ than WT (*p* < 0.01) in both basal conditions as well as following hCG stimulation (Figure 3L), confirming that i) *Ankrd31* ^*−/−*^ Leydig cells possessed an impaired ability to respond to LH stimulation and ii) the deregulation of Kp/KISS1R system and of steroidogenesis modulators negatively affected TT production in *Ankrd31* ^*−/−*^ mice.

### Kp-10 Enhances KISS1R–ANKRD31 Protein Interaction and Actin Polymerization in Leydig Cells

In order to investigate if a stimulation of KISS1R may be able to regulate Leydig cell gene expression, *via* the cytoskeletal–nucleoskeletal pathway, IP experiments were carried out in murine primary Leydig cell cultures to show a physical interaction among KISS1R, ANKRD31, and F-actin.

**FIGURE 4:**
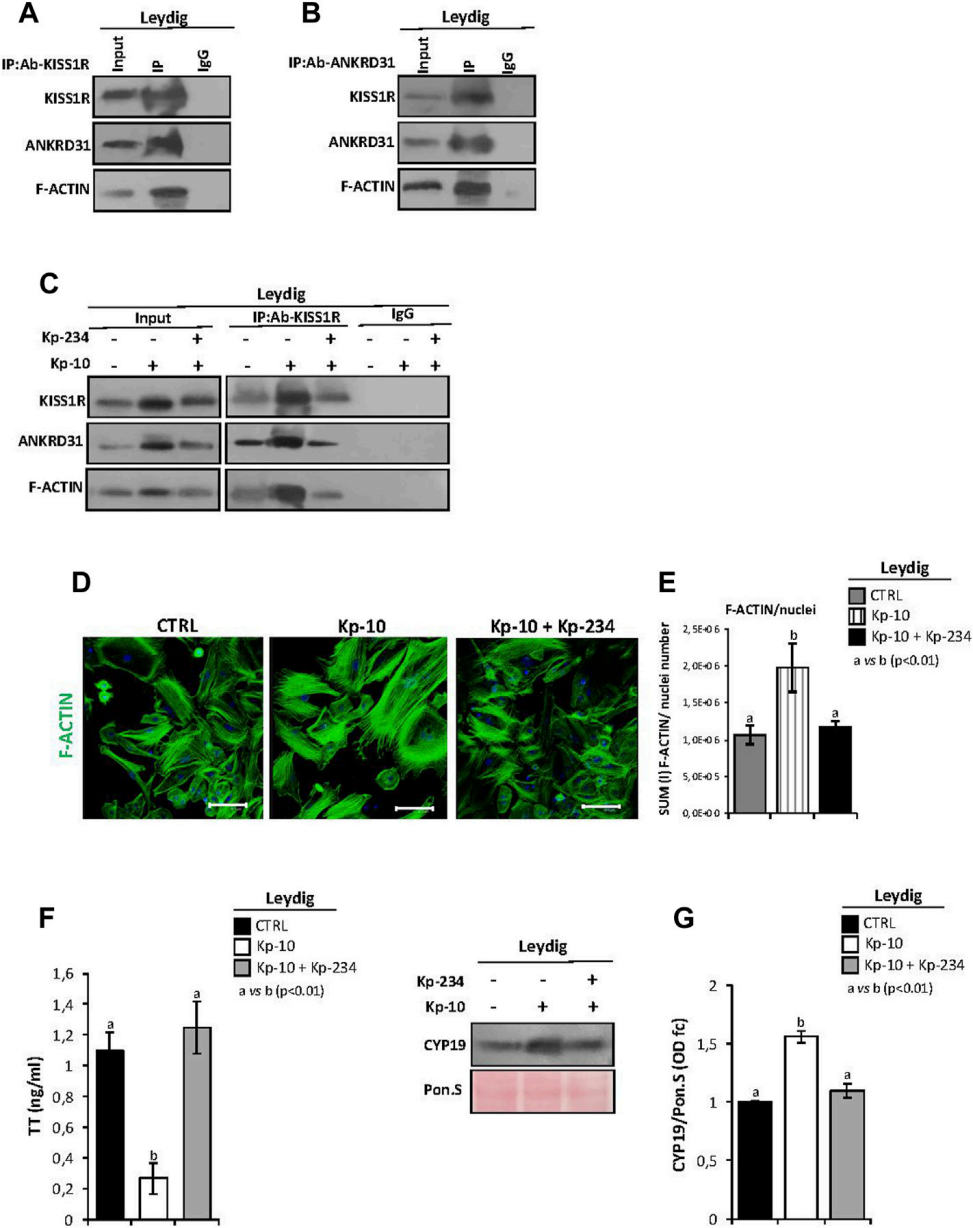
**(A,B)** IP in murine primary Leydig cells. Total proteins collected from murine primary Leydig cell cultures were immunoprecipitated using **(A)** KISS1R and **(B)** ANKRD31 antibodies, respectively. Protein interaction among KISS1R, ANKRD31, and F-actin was detected by Western blot analysis. **(C)** IP in murine primary Leydig cells *in vitro* treated with Kp-10 alone (0.1 µM) or in combination with the specific antagonist Kp-234 (1 µM) using KISS1R antibody. Protein interaction among KISS1R, ANKRD31, and F-actin was detected by Western blot analysis. **(D)** Immunofluorescence analysis of F-actin, using phalloidin staining (green) in murine primary Leydig cells *in vitro* treated with Kp-10 alone (0.1 µM) or in combination with the specific antagonist Kp-234 (1 µM). Nuclei were labeled with TO-PRO3 iodide (blue). Scale bar: 37.5 µm. **(E)** Quantitative immunofluorescence analysis: F-actin signals were normalized against nuclei number, expressed in SUM(I) values and reported as mean ± SEM; experimental groups with statistically significant differences (*p* < 0.01) were indicated with different letters; the experimental groups without statistically significant differences were indicated with the same letter. **(F)** Analysis of TT content (as ng/ml) in culture media of Leydig cells *in vitro* treated with Kp-10 alone (0.1 µM) or in combination with the specific antagonist Kp-234 (1 µM). All the data were reported as mean ± SEM; **p < 0.01. Experimental groups with statistically significant differences (*p* < 0.01) were indicated with different letters. **(G)** Western blot analysis of CYP19 in murine primary Leydig cells *in vitro* treated with Kp-10 alone (0.1 µM) or in combination with the specific antagonist Kp-234 (1 µM). Signals were quantified by the densitometry analysis and normalized to Ponceau Red (Pon.S). Data are expressed in O.D. values as fold change (O.D. fc), relatively to the CTRL group, and reported as mean ± SEM; experimental groups with statistically significant differences (*p* < 0.01) were indicated with different letters.

We first immunoprecipitated KISS1R from total proteins extracted from Leydig cells (IP-KISS1R), followed by immunoblotting with KISS1R, ANKRD31, and F-actin antibodies (Figure 4A). The results showed stronger KISS1R, ANKRD31, and F-actin signals in IP-KISS1R as compared with a significantly weaker control signals, suggesting a protein complex formation among KISS1R, ANKRD31, and F-actin in Leydig cells (Figure 4A). Accordingly, the immunoprecipitation of ANKRD31 (IP-ANKRD31) from total proteins extracted from Leydig cells and the immunoblotting with KISS1R, ANKRD31, and F-actin antibodies confirmed the existence of the protein complex (Figure 4B).

In addition, murine primary Leydig cell cultures were *in vitro* treated with Kp-10 alone (0.1 µM) or in combination with the specific antagonist Kp-234 (1 µM), with the aim to investigate the cytoskeletal–nucleoskeletal responsiveness to the Kp/KISS1R system activation in Leydig cells. Following the treatment, total Leydig cell protein extracts, derived from the three experimental groups (CTRL, Kp-10, and Kp-10+Kp-234), were used for KISS1R protein IP experiments. Immunoblotting with KISS1R, ANKRD31, and F-actin antibodies showed KISS1R, ANKRD31, and F-actin signals in IP-KISS1R carried out in all experimental groups (Figure 4C). Interestingly, results evidenced a strong increase of protein interaction among KISS1R, ANKRD31, and F-actin following Kp-10 treatment (Figure 4C). This increase was significantly counteracted by Kp-234 treatment and was dependent on the variations of total protein content, as confirmed by the analysis of input samples (total lysates isolated before the IP) (Figure 4C). Data suggested that Kp effectively increases the expression of KISS1R, ANKRD31, and F-actin and, consequentially, the formation of protein complex in Leydig cells.

Phalloidin staining was carried out in Leydig cells *in vitro* treated with Kp-10 alone or in combination with the specific antagonist Kp-234, in order to investigate the effect of Kp/KISS1R system activation on actin polymerization (Figure 4D). As showed, relatively to the CTRL group, a strong increase in phalloidin staining was observed in Leydig cells treated with Kp-10, whereas the signal intensity in the combined treatment Kp-10 + Kp-234 was similar to the control (Figure 4D). Consistently, phalloidin immunofluorescence quantification, relative to the CTRL group, confirmed a significant increase of the signal in Leydig cells treated with Kp-10 (*p* < 0.01); this effect was efficiently counteracted by Kp-234 (Figure 4E), suggesting that the Kp/KISS1R system activation in Leydig cells promoted an active F-actin synthesis, in addition to the enhancing of the protein complex among KISS1R, ANKRD31, and F-actin.

In order to show if KISS1R, ANKRD31, and F-actin protein complex may affect Leydig cell biological functions, TT levels were quantified in culture media of Leydig cells *in vitro* treated with Kp-10 alone (0.1 µM) or in combination with Kp-234 (1 µM) by the LC-MS/MS analysis. Interestingly, TT levels significantly decreased following Kp-10 treatment (*p* < 0.01), relatively to the CTRL group, and such an effect was significantly counteracted by Kp-234 treatment (Figure 4F). In addition, a significant increase of CYP19 protein content was observed after Kp-10 treatment (*p* < 0.01), in comparison to the CTRL group, by Western blot (Figure 4G). This effect was counteracted by Kp-234 (Figure 4G), suggesting that Kp/KISS1R system activation in Leydig cells may enhance testosterone conversion to estrogens.

### Kp-10 Enhances Leydig Cell Gene Expression *via* the Cytoskeletal-Nucleoskeletal Pathway

**FIGURE 5:**
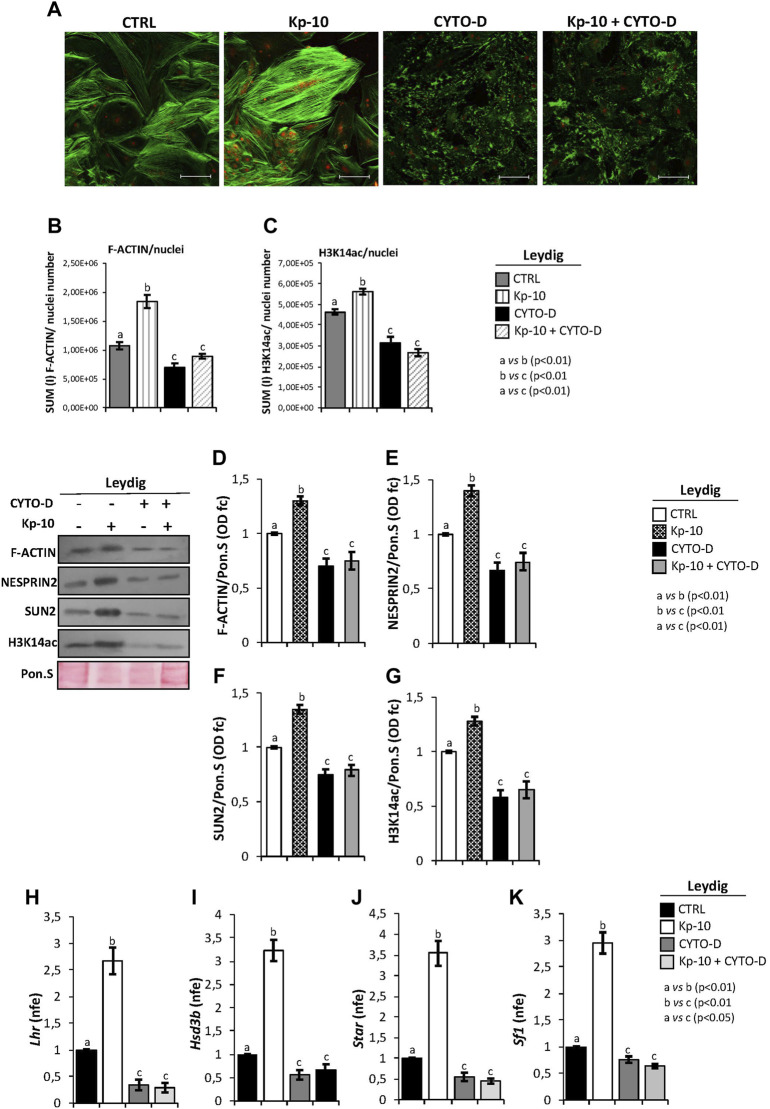
**(A)** Immunofluorescence analysis of F-actin (green) and H3K14ac (red) in murine primary Leydig cells *in vitro* treated with Kp-10 (0.1 µM) and cytochalasin D (CYTO-D) (10 µM) alone or in combination with Kp-10 (Kp-10+CYTO-D). Nuclei were labeled with TO-PRO3 iodide (blue). Scale bar: 37.5 µm. **(B,C)** Quantitative immunofluorescence analysis of F-actin and H3K14ac signals. Data were normalized against nuclei number, expressed in SUM(I) values and reported as mean ± SEM; experimental groups with statistically significant differences (*p* < 0.01) were indicated with different letters; the experimental groups without statistically significant differences were indicated with the same letter. Western blot analysis of **(D)** F-actin, **(E)** NESPRIN2, **(F)** SUN2, and **(G)** H3K14ac proteins levels in murine primary Leydig cells *in vitro* treated with Kp-10 (0.1 µM) and cytochalasin D (CYTO-D) (10 µM) alone or in combination with Kp-10 (Kp-10 + CYTO-D). Signals were quantified by the densitometry analysis and normalized to Ponceau Red (Pon.S). Data are expressed in O.D. values as fold change (O.D. fc), relatively to the CTRL group, and reported as mean ± SEM; experimental groups with statistically significant differences (*p* < 0.01) were indicated with different letters; the experimental groups without statistically significant differences were indicated with the same letter. The differential expression analysis of Leydig cell genes in primary Leydig cells *in vitro* treated with Kp-10 (0.1 µM) and cytochalasin D (CYTO-D) (10 µM) alone or in combination with Kp-10 (Kp-10+CYTO-D), by qRT-PCR. **(H)** *Lhr*, **(I)** *Hsd3b*, **(J)** *Star*, and **(K)** *Sf1* expression levels were normalized using *Rp18S* as a housekeeping gene and expressed as normalized fold expression (n.f.e.), relatively to the CTRL group. All data are reported as mean value ± S.E.M; experimental groups with statistically significant differences (*p* < 0.01) were indicated with different letters; the experimental groups without statistically significant differences were indicated with the same letter.

With the purpose to demonstrate that Kps could regulate gene expression in Leydig cells, *via* the activation of the cytoskeletal–nucleoskeletal pathway, murine primary Leydig cell cultures were *in vitro* treated with Kp-10 (0.1 µM), cytochalasin D (10 µM), and, finally, with Kp-10 in combination with cytochalasin D. Following pharmacological treatments, co-immunofluorescence analysis was carried out to investigate the responsiveness of F-actin and H3K14ac to the different experimental conditions (Figure 5A).

Accordingly, Kp-10 induced a strong increase of F-actin signal, as previously reported, whereas a complete disaggregation of F-actin was observed following cytochalasin D treatment. In addition, Kp-10 was not able to effectively counteract the depolymerization effect of cytochalasin D on F-actin filaments (Figure 5A). Interestingly, H3K14ac immunofluorescence signal was increased in Leydig cells treated with Kp-10, whereas a weak signal was detected in Leydig cells treated with cytochalasin D alone or in combination with Kp-10 (Figure 5A).

Immunofluorescence quantification data, relatively to the CTRL group, confirmed the significant increase of F-actin staining in Leydig cells treated with Kp-10 (*p* < 0.01) and its reduction after cytochalasin D alone or in combination with Kp-10 (*p* < 0.01) (Figure 5B). In addition, H3K14ac immunofluorescence quantification showed the same trend observed for F-actin. In details, a significant increase of H3K14ac amount was observed in Leydig cells treated with Kp-10 (*p* < 0.01) and, consistently, a strong reduction occurred when Leydig cells were treated with cytochalasin D alone or in combination with Kp-10 (*p* < 0.01) (Figure 5C), suggesting that Kp/KISS1R system activation modulates the epigenetic landscape of Leydig cells *via* F-actin synthesis.

To strongly demonstrate this hypothesis, Western blot analysis of F-actin, cytoskeletal–nucleoskeletal mediators, as NESPRIN2 and SUN2, and H3K14ac was carried out in murine primary Leydig cells *in vitro* treated with Kp-10 and cytochalasin D alone or in combination with Kp. For F-actin, results showed a significant increase following Kp-10 treatment (*p* < 0.01) and a significant reduction following cytochalasin D treatment alone or in combination with Kp-10 (*p* < 0.01), in comparison with the CTRL group (Figure 5D). In addition, both NESPRIN2 and SUN2 showed higher protein levels following Kp-10 treatment and lower protein levels following cytochalasin D treatment alone or in combination with Kp-10, than the CTRL group, respectively (*p* < 0.01) (Figures 5E,F). Finally, relatively to the CTRL group, a significant increase of H3K14ac protein levels was observed in Leydig cells treated with Kp-10 (*p* < 0.01), and a significant reduction was observed when Leydig cells were treated with cytochalasin D alone or in combination with Kp-10 (*p* < 0.01) (Figure 5G).

With the aim to highlight that Kps, *via* the cytoskeletal–nucleoskeletal pathway activation, could enhance H3K14ac and, in turn, Leydig gene expression, the expression analysis of several markers was carried out by qRT-PCR. The results showed a significant increase of *Lhr*, *Hsd3b*, *Star*, and *Sf1* expression levels following Kp-10 treatment (*p* < 0.01), in comparison with the CTRL group (Figures 5H–K), whereas a significant reduction was observed following cytochalasin D treatment, alone or in combination with Kp-10 (*p* < 0.01) (Figures 5H–K), suggesting that Kps enhance Leydig cell gene expression increasing key modulators of the cytoskeletal–nucleoskeletal pathway and, then, epigenetic markers.

## Discussion

The role of Kp/KISS1R system in the testis, especially in Leydig cells, has been deeply suggested (Anjum et al., 2012; Chianese et al., 2013, 2015, 2017; Salehi et al., 2015; Han et al., 2020; Meccariello et al., 2020; Gloria et al., 2021).

Important biological processes including cytoarchitecture organization, chromatin remodeling, and the optimal maintenance of male fertility are under the control of ANKRD proteins (Cunhaa and Mohler, 2009; Argudo et al., 2019; Tang et al., 2020; Manfrevola et al., 2021a). In this scenario, particular attention has been given to ANKRD31, a key regulator of male meiosis and epididymal sperm maturation (Boekhout et al., 2019; Acquaviva et al., 2020; Manfrevola et al., 2021b). Despite a clear effect of Kp/KISS1R signaling on ANKRD expression has been demonstrated in GnRH neurons, a possible crosstalk or a functional interaction among the Kp/KISS1R system and ANKRDs, outside the HPG axis, as well as in Leydig cells remain unknown (Soga et al., 2016). Hence, in this work, we have characterized the testicular responsiveness to Kp/KISS1R system activation, defining its role in Leydig cell physiology, *via* ANKRD31 and the cytoskeletal–nucleoskeletal pathway.

First, we confirmed the expression of the Kp/KISS1R system in murine Leydig cells. Then, we showed that Kp-10 *in vitro* treatment exerted a testicular effect on KISS1R, promoting both mRNA and protein expression. Analogously, Kp-10 treatment significantly stimulated the expression of ANKRD31, SPECTRIN, β-actin, and F-actin. A similar effect was observed in GnRH neurons where Kp signaling increased ANKRD26 gene expression (Soga et al., 2016). ANKRDs, acting as a scaffold useful for protein–protein interactions, may regulate complex cellular mechanisms *via* the cytoskeletal–nucleoskeletal pathway. Indeed, Kp inhibits GnRH neuronal movement through an intracellular mechanotransduction pathway, in turn dependent on ANKRD26 expression (Soga et al., 2016). These data lead us to hypothesize that a similar signaling can also occur in the testis. Actually, Kp-10 increased NESPRIN2 and SUN2 protein levels, the two fundamental actors of the linker of nucleoskeleton and cytoskeleton (LINC) complex (Crisp et al., 2006; Rajgor and Shanahan, 2013; Guilluy et al., 2014; Manfrevola et al., 2021a). However, in Kp-10 treatment, the mRNA and protein levels of NESPRIN2 and SUN2 were not perfectly correlated with each other probably due to other levels of regulation between transcript and protein products (Maier et al., 2009; Vogel and Marcotte, 2012).

Considering that our results confirmed the expression of the Kp/KISS1R system in Leydig cells, we wanted to assess i) the Kp-dependent cytoskeletal–nucleoskeletal pathway switch-on in Leydig cells and ii) the potential effect of Kp-10 on the expression levels of Leydig cell markers. With this in mind, we evaluated an increase in gene expression of *Lhr*, *Hsd3b*, *Star,* and finally *Sf1*, induced by Kp-10*.* As well known, mechanical forces could regulate gene expression through the modulation of nuclear chromatin and epigenetic landscapes, *via* the cytoskeletal–nucleoskeletal pathway (Li et al., 2020; Manfrevola et al., 2021a). Interestingly, testicular Kp-10 treatment was associated with a strong increase of H3K14ac, in correlation with a decrease of several HDACs, including HDAC1, HDAC2, and HDAC4. The epigenetic histone modification H3K14ac is defined as a canonical marker of active gene expression able to modulate chromatin folding in favor of transcriptional activation; furthermore, it participates to several stages of spermatogenesis and male germ cell maturation (Chioccarelli et al., 2020). It is of fundamental interest to specify that a dynamic interplay between histone H3 acetylation and HDAC activity in Leydig cells has been well reported. Indeed, studies on the LC540 Leydig cell *in vitro* system have demonstrated that HDAC inhibition increased histone H3 acetylation promoting the gene expression of steroidogenic markers *Star*, *Hsd3b*, and *Hsd17b* (Sadasivam et al., 2015). In agreement, H3K14ac acetylation in *Star* promoter regulates its gene expression in rat testes (Liang et al., 2012). Based on these intriguing observations, we suppose that Kp-10 enhances the expression of Leydig cell markers regulating histone acetylation, through the cytoskeletal–nucleoskeletal pathway activated by the KISS1R–ANKRD31 interaction.

To assess our hypothesis, we carried out several histological and molecular analyses in *Ankrd31* ^*−/−*^ testis, an experimental model useful to verify the responsiveness of the Kp/KISS1R system, and in turn of the Leydig cell functions, dependent on ANKRD31 loss. Interestingly, in *Ankrd31* ^*−/−*^ testis, a complete morphological deregulation of Leydig cells, associated with a severe Kp/KISS1R system disruption, was observed. Consistently, the observed reduction of F-actin and H3K14ac, in association with the impaired LHR, HSD3β, STAR, and SF1 expression levels, as well as the affected testosterone secretion, strongly confirmed that the lack of ANKRD31 negatively influenced the Kp/KISS1R system and, in turn, the cytoskeletal–nucleoskeletal actors needful for Leydig cell functions.

In order to deeply demonstrate the fundamental role of the KISS1R–ANKRD31 interaction in the enhancing of Leydig cell functions *via* the cytoskeletal–nucleoskeletal pathway, we isolated murine primary Leydig cells and showed, for the first time, a protein interaction among KISS1R, ANKRD31, and F-actin proteins. Then, we treated primary Leydig cell culture with Kp-10 alone or in combination with the specific antagonist of KISS1R, Kp-234, in order to evaluate their biological effects on such an interaction. Interestingly, Kp-10 induced the expression of KISS1R and ANKRD31, promoting not only the protein interaction between them but also, surprisingly, a strong F-actin synthesis. Kp-234 reverted the Kp-dependent increase. In support to IP experiments, phalloidin staining clearly showed a strong increase of the signal after Kp-10 stimulation in Leydig cells; this effect was counteracted when Leydig cells were treated with Kp-10 in combination with Kp-234. The responsiveness of Leydig cells to Kp-10 stimulation is in accordance with previous studies that showed i) the expression of Kp system, including KISS1R, in Leydig cells and ii) the increase of Kiss1/KISS1R expression in Leydig cells following Kp-10 treatment (Han et al., 2020). In addition, the reduction of TT levels following Kp-10 stimulation, associated with CYP19 increase, strongly suggested an enhanced testosterone-to-estrogen conversion, thus confirming the primary role of the Kp/KISS1R system in the regulation of Leydig cell biological functions. These data are consistent with our previous study carried out in anuran amphibian, where low doses of Kp-10 affected intratesticular TT levels in favor of its estrogen conversion, due to CYP19 increase. Interestingly, there as here, Kp-10 modulation of steroidogenesis may be suggested to be dose-dependent (Chianese et al., 2017).

At this point, we wondered if the induction of the cytoskeletal–nucleoskeletal pathway, Kp-dependent, could be responsible for an epigenetic modulation able to favor the gene expression of Leydig cell markers. To answer this question, we treated murine primary Leydig cell cultures with Kp-10, cytochalasin D, a potent F-actin depolymerization factor, and with a combination of both. Consistent with our previous data, Kp-10 induced a massive F-actin synthesis in association with increased NESPRIN2, SUN2, and H3K14ac levels. As expected, a significant reduction of F-actin synthesis occurred following cytochalasin D treatment. More interestingly, a reduction of NESPRIN2, SUN2, and H3K14ac levels was induced by cytochalasin D, an effect also displayed in the combined treatment with Kp-10.

The observed increase in the gene expression of Leydig cell markers *Lhr*, *Hsd3b*, *Star*, and *Sf1*, following Kp-10 treatment, and their respective reduction following the treatment with cytochalasin D alone or in combination with Kp-10, could be strongly dependent on H3K14ac levels, thus to conclude that the ignition of the mechanostraduction pathway, Kp-dependent, regulates the epigenetic landscape of Leydig cells, thus favoring active gene expression.

NESPRIN and SUN proteins are implicated in the organization of nuclear envelope, in the interaction of nucleus with cytoskeletal filaments and, finally, in the chromatin remodeling (Manfrevola et al., 2021a). In addition, the participation of NESPRIN2 and SUN2 in the mechanotransduction pathways useful for the regulation of histone posttranslational modifications has also been reported (Kandert et al., 2007; Camozzi et al., 2012; Alam et al., 2016). In this regard, fibroblast cells harboring *Lmna* gene mutations showed alteration in the NESPRIN2 localization and in the distribution of both phosphorylated RNA polymerase II and acetylated histones, with affected chromatin topology and transcriptional activity (Kandert et al., 2007). In the same scenario, skin fibroblasts harboring *Lmna* gene mutations showed a direct correlation between SUN2 mislocalization and the loss of histone methylation (Camozzi et al., 2012). A similar association among the NESPRIN2–SUN2 complex and histone H3 acetylation is not to be excluded in Leydig cells, as our data suggest.

Taken together, our results demonstrate how Kp/KISS1R system activation in Leydig cells is able to induce KISS1R/ANKRD31 interaction, downstream triggering the activation of the cytoskeletal–nucleoskeletal pathway. Meanwhile, we have showed the responsiveness of the epigenetic signature to the cytoskeletal–nucleoskeletal pathway, resulting in the switch-on of Leydig cell gene expression.

## Data Availability

The raw data supporting the conclusions of this article will be made available by the authors, without undue reservation.
